# Mental health among Sweden Finns: A systematic scoping review

**DOI:** 10.1177/13634615251379430

**Published:** 2025-10-25

**Authors:** Mattias Strand, Mona Lindqvist

**Affiliations:** 1 Centre for Psychiatry Research, Department of Clinical Neuroscience, 27106 Karolinska Institutet; 2 Transcultural Centre, Stockholm Health Care Services, Region Stockholm

**Keywords:** minority health, labor migration, diaspora, cultural competence, structural competence

## Abstract

Sweden Finns are one of five officially recognized national minority groups in Sweden. As a group, Sweden Finns have been socioeconomically underprivileged in comparison with the Swedish majority population, and tend to be worse off in terms of somatic health. However, the literature on mental health among Sweden Finns has not previously been systematically appraised. The aim of this scoping review was to synthesize the available evidence on mental health among Sweden Finns or, when the minority terminology is not used in the literature, among individuals in Sweden with a Finnish background (including the so-called Finnish war children). Systematic literature searches in MEDLINE, Web of Science, and PsychINFO were performed. A total of 46 publications met the inclusion criteria; however, very few made use of the self-identification principle central to official Swedish minority politics. The synthesized evidence shows that Sweden Finns tend to be worse off in terms of mental health compared with the Swedish-born majority population, even after adjusting for socioeconomic factors. They are more often diagnosed with various serious psychiatric disorders, such as schizophrenia and other psychotic disorders. Alcohol and other substance-use disorders are also more common. Moreover, substantially higher suicide rates are consistently reported in this group, although no recent studies exist. The few published qualitative studies emphasize the importance of cultural competence in the care of elderly Sweden Finns with neurocognitive deficits. More qualitative and ethnographically oriented research is needed to explore causal pathways behind the observed patterns and to guide potential preventive measures.

## Introduction

Sweden Finns are one of five officially recognized national minority groups in Sweden, alongside the Sámi, Tornedalians, Romani, and Jews. Their national minority status is based on the far-reaching historical roots of Finns and the Finnish language in Sweden (Statens offentliga utredningar, 2017a), largely because what is now Finland was part of the Swedish nation until 1809. The 16th and 17th centuries saw large-scale migration of so-called Forest Finns from what was then the eastern part of the nation to crown lands in the mid-western region of Sweden proper, where they expanded the arable land by practicing slash-and-burn agriculture on uncultivated forest terrain. This historical Finnish migrant population has since gradually become fully assimilated through intermarriage with the Swedish majority population and has lost the Finnish language. Today's Finnish-speaking population in Sweden mostly migrated to Sweden in the modern era. In general, three waves of more recent migration from Finland to Sweden can be outlined:

- The approximately 70,000 so-called Finnish war children who were evacuated to Sweden during the Winter War and Continuation War 1939–1944 for humanitarian reasons, of whom around 7100 were subsequently adopted by their foster families and remained in the country (Mattsson, 2018).^1^
- The large population of Finnish migrant workers who primarily came to Sweden during the 1960s and 1970s, an era in which more than 300,000 Finns were recruited to meet the large needs of the Swedish post-war industrial sector (Borg, 2016; Weckström, 2016). Although the question of Swedish neutrality during the Second World War remains a subject of debate, Sweden was largely unharmed by the war, whereas war-torn Finland was plagued by destruction, poverty, and unemployment. The prospects for young Finns were bleak and moving to Sweden for work was seen by many as an exciting opportunity (Liimatainen, 2022; Lukkarinen Kvist, 2006). These Finnish migrants were typically young, childless, and relatively poorly educated. Most of them found employment in the industrial and manufacturing sectors, but working as janitors, nursing assistants, or in restaurants was also common (Liimatainen, 2022). When the Finnish economy subsequently bloomed in the 1970s and 1980s, some chose to return but many ultimately remained in Sweden (Lukkarinen Kvist, 2006).
- The more educated Finns, as a group—sometimes referred to as “the Nokia generation”, reflecting the rise of Finland as an economically and technically highly advanced country—who moved to Sweden from the 1980s and onwards to study and work (Lukkarinen Kvist, 2006).

Registration according to ethnicity is generally prohibited by Swedish law and the exact number of Sweden Finns in Sweden is therefore difficult to assess. The number of individuals in Sweden who are themselves born in Finland or who have at least one Finnish-born parent was 387,000 in 2023 (Statistics Sweden, 2024). However, as discussed in more detail below, this is not necessarily the same as belonging to the national minority of Sweden Finns. Up until 2016, people born in Finland were the largest immigrant group in Sweden (since when Syrian migrants have been more numerous) (Statistics Sweden, 2023). Today, a large proportion of Sweden Finns lives in the Stockholm area or in historically industrial cities around Lake Mälaren, such as Eskilstuna, Västerås, and Södertälje, which means that the Sweden Finn minority is a largely urban population (Statens offentliga utredningar, 2017b).

### Who is a Sweden Finn?

It is important to distinguish between the various terms for individuals with a Finnish background in the literature, although the English language lacks some of these distinctions. Finland has two official languages: Finnish and Swedish. The population of Finland can be divided into the Finnish-speaking majority population (Finnish: suomalaiset, Swedish: finnar) and the Swedish-speaking minority population (Finnish: suomenruotsalaiset, Swedish: finlandssvenskar; approximately 5% of the Finnish population). Finns living in Sweden can have either Finnish or Swedish as their mother tongue, whereas the term Sweden Finns (Finnish: ruotsinsuomalaiset, Swedish: sverigefinnar) specifically refers to Finnish-speaking residents in Sweden and their offspring (who may or may not speak Finnish). The broader term “Finnish background” can refer to any of these groups. However, because of considerable intermarriage, and the fact that the Swedish national minority legislation explicitly makes use of a self-identification principle rather than any objective criteria in deciding who belongs to a national minority group such as the Sweden Finns, the borders between the groups outlined above are not definite.

Many of the rights associated with national minority status concern the minority languages. For example, Sweden Finns have the explicit right to use the Finnish language when communicating with governmental, regional, and municipal authorities and agencies (Statens offentliga utredningar, 2020). Even so, national minority status as such is not defined by language proficiency. Historically, the national minorities have often had to struggle for their right to use their minority languages in everyday life (Lainio, 2014) and parents may have felt pressured to abstain from teaching children their own language out of fear of stigmatization and discrimination (Rasmussen & Nolan, 2011; Weckström, 2016). It is therefore fully possible to self-identify as a Sweden Finn without actually mastering the Finnish language—this is fairly common among second- and third-generation Sweden Finns (Statens offentliga utredningar, 2017a). Moreover, the Finnish war children who remained in Sweden were often adopted into Swedish-speaking families at a young age and have tended to gradually lose their native Finnish language over time (Lagnebro, 1994). There is no strict relationship between a certain national minority individual and the minority language usually associated with the group—e.g., Finnish Roma often speak Finnish rather than Romani chib and Sámi people in the Torne River Valley may very well have Meänkieli, the official minority language of the Tornedalians, as their mother tongue (Statens offentliga utredningar, 2017a).

Furthermore, the self-identification principle implies that it is fully possible to identify as belonging to several national minorities at once—e.g., Sweden Finn and Sámi—or, if one's parents migrated to Sweden from different countries, as both Sweden Finn and Chilean Swede, for example. Naturally, people in Sweden with a Finnish background may also view themselves as Swedes, Finns, and Sweden Finns all at once. Finally, the self-identification principle means that any individual is free to view themselves as not belonging to one of the national minorities, regardless of mother tongue and origin.

Over the years, a number of research studies have examined the health status—somatic as well as mental health—of Sweden Finns and other groups in Sweden with a Finnish background. Like other migrant worker groups, Sweden Finns have often been socioeconomically underprivileged in comparison with the Swedish majority population (although this is not necessarily the case for all Finnish migrants to Sweden), which tends to have a negative impact on health. It is well established that individuals with a Finnish background in Sweden tend to be worse off in terms of somatic health compared with the rest of population. For example, Finnish-born women and men in Sweden, as well as women and men in the so-called “second generation” born in Sweden with at least one Finnish-born parent, display clearly elevated total mortality rates compared with the Swedish-born population and their offspring (Manhica et al., 2015; Östergren et al., 2023). These elevated rates are particularly notable considering that most other migrant groups to Sweden actually display lower total mortality rates than the Swedish-born population (Wallace, 2022) (although the rates for non-Nordic migrant groups may be falsely low because of so-called salmon bias; Dunlavy et al., 2022). Moreover, cardiovascular disease, cerebrovascular accidents, diabetes mellitus, and obesity are more common among women and men in Sweden with Finnish background compared with the rest of the population (Albin et al., 2014; Carlsson et al., 2008, 2014; Li et al., 2013; K. Sundquist & Li, 2006). To the best of our knowledge, however, the literature on mental health and illness among Sweden Finns and/or people with a Finnish background in Sweden has not previously been assessed and synthesized in a systematic way.

### Aim and Objectives

The aim of this systematic scoping review is to assess and synthesize the available evidence on mental health among Sweden Finns or, when this distinction is not explicitly made in the literature, among individuals in Sweden with a Finnish background. The main objectives are to systematically review how Sweden Finns differ from the rest of the population in terms of mental health and how they describe their experiences and needs related to mental health and healthcare. Here, it is important to underscore once more that, as described above, belonging to the national minority of Sweden Finns (or any of the other national minorities in Sweden) is based on a self-identification principle. Thus, there are no firm objective criteria that can be applied in determining who is a Sweden Finn and who is not when, for example, conducting an epidemiological register study. Furthermore, although Swedish national population registers are generally considered as being of very high quality, registering people based on ethnicity and/or race is largely prohibited according to Swedish law. In this scoping review, we therefore include studies that explicitly involve a study population of Sweden Finns (participants who have actually been asked whether they see themselves as belonging to the national minority) as well as studies that involve Finns living in Sweden; first-, second-, or third-generation Finnish immigrants to Sweden; or Finnish war children evacuated to Sweden during the Winter War and Continuation War, even though we cannot be certain that all individual members of these groups view themselves as Sweden Finns. It should also be noted that terms such as “second-generation” and “third-generation” migrants have been rightly criticized as imprecise and conceptually flawed (Borrelli & Ruedin, 2024); nevertheless, since they are very common in the research literature under review, we occasionally use them here.

## Methods

### Eligibility Criteria

Unlike regular systematic reviews, the aim of a systematic scoping review is not to answer a narrowly specified research question (such as “what is the prevalence of schizophrenia among Sweden Finns?”) but to map and clarify the conceptual boundaries of a heterogeneous research field that may not yet be ripe for a more precise systematic review. Thus, a systematic scoping review usually engages with a broader literature covering a variety of research methodologies. This is reflected in the eligibility criteria outlined below. To be eligible for the current systematic scoping review, research publications should:

- Concern and/or involve Sweden Finns; Finns living in Sweden; first-, second-, or third-generation Finnish immigrants to Sweden; or Finnish war children evacuated to Sweden during the Winter War and Continuation War.
- Concern the topic of mental health (including substance-use disorders and neurocognitive disorders).
- Have been published in the past 30 years; i.e., between 1994 and 2023 (as described below, an updated search for 2024 was also conducted). Although it might also be interesting to analyze longer historical patterns, in this scoping review we wish to mainly focus on contemporary trends in mental health. It can also be noted that in 1995, Sweden signed the Council of Europe's Framework Convention for the Protection of National Minorities, which subsequently lead to the formal recognition of Sweden Finns as a national minority in Sweden; thus, the time frame used coincides with the broader emergence of Sweden Finns as a labeled social category.

Furthermore, the systematic scoping review did not include:

- Studies that only compare Finns in Finland with Swedes in Sweden, or studies that compare the Finnish-speaking and Swedish-speaking populations of Finland.
- Studies that solely concern social determinants of health, such as unemployment or education levels, that do not specifically report mental health outcomes.
- Essays, novels, autobiographies, or popular science texts, of which there is a rich literature concerning Sweden Finns and Finnish war children (see, for example, the English-language volume edited by Saffle, 2015), which is beyond the scope of this review.
- Single articles that are already included in the review in the form of a broader doctoral thesis.

No restrictions regarding publication language were imposed.

### Data Sources and Search Strategy

Systematic searches in the databases MEDLINE, Web of Science, and PsychINFO were performed, using the following search string: (finn* AND swed*) OR (“finnish war”) OR (finland AND “world war”). Several more narrow search strings were initially tested, but because they did not manage to capture a number of known articles relevant to the topic, a broader search string was necessary. Furthermore, reference lists of included studies were hand-searched to identify additional studies of relevance. The main database search was conducted in April 2023, with additional search updates in September 2023 and April 2024. Relevant trial registries (ClinicalTrials.gov, the WHO International Clinical Trials Registry Platform, the EU Clinical Trials Register, and the Open Science Framework) were also searched for signs of preregistered studies that have not been published within reasonable time.

### Data Selection, Collection, and Synthesis

**Figure 1.**
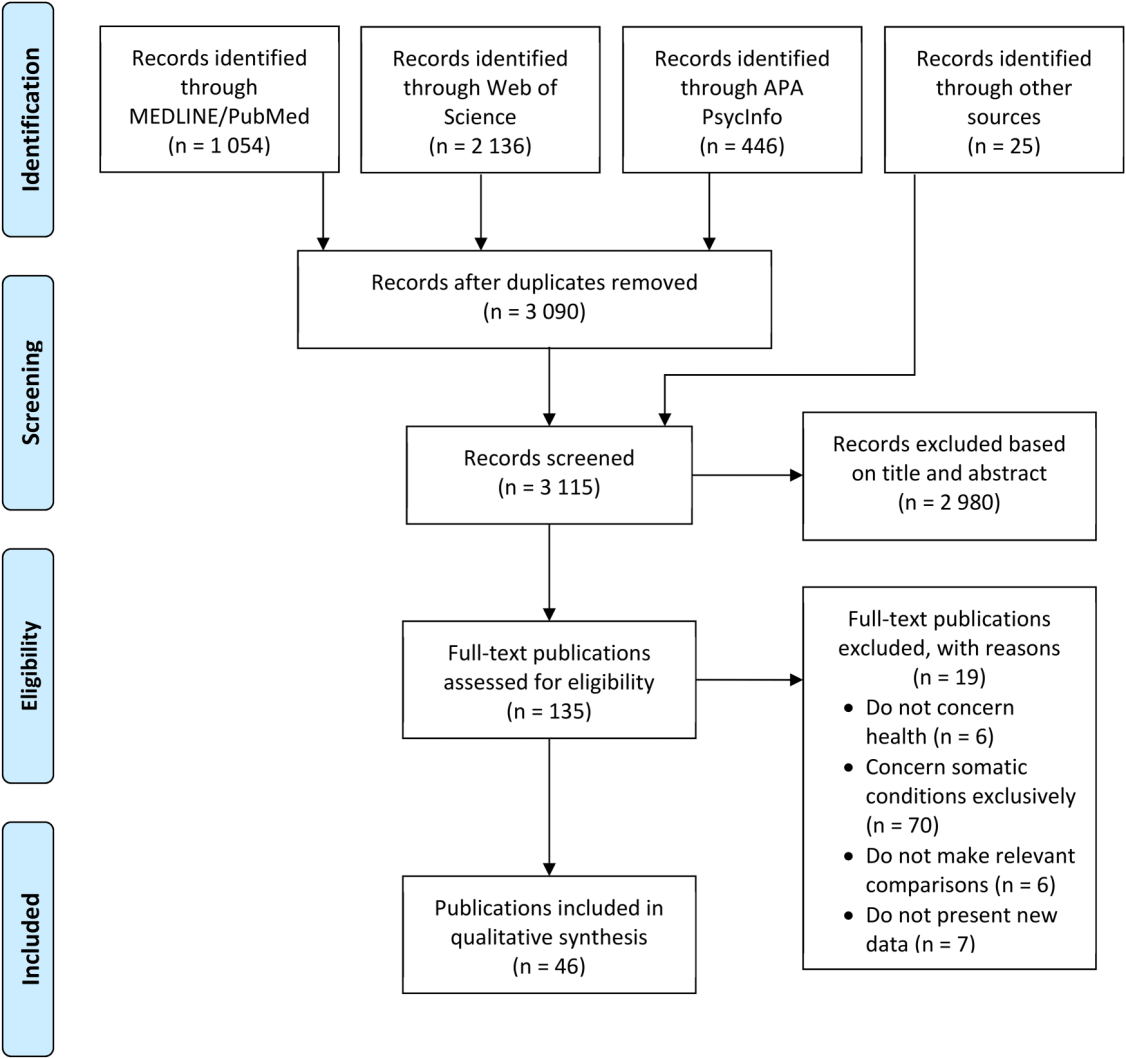
PRISMA Flow Chart Outlining the Data Selection Procedure.

First, all titles/abstracts were scanned and relevant papers for further full-text assessment were selected. Second, all full-text papers of potential relevance were assessed, to identify papers for inclusion in the systematic scoping review. The data selection procedure is illustrated in the form of a PRISMA flow chart in Figure 1. A data collection sheet including relevant study aspects, such as publication year, methodology, sample, and findings, was created and data were then extracted from the original studies.

As stated above, the data synthesis in this scoping review did not focus on single numerical outcome measures (such as suicide rates or odds ratios for psychotic disorders). Instead, the aim of the synthesis was to identify overall patterns and trends in the existing literature on mental health among Sweden Finns.

Publication bias was assessed through searching for preregistered but unpublished studies, as described above. Apart from this, no systematic assessments of methodological rigor or bias were performed, because of the multifaceted nature of the study types included in a scoping review.

This systematic scoping review was performed in accordance with the guidelines for scoping reviews developed by the Joanna Briggs Institute (Peters et al., 2015). As far as possible, we have also adhered to the PRISMA statement guidelines for systematic reviews (Liberati et al., 2009), although not all checklist items are fully applicable to scoping reviews. To ensure maximum procedural transparency, the review protocol was preregistered on the Open Science Framework (https://osf.io/zm6y8).

## Results

In total, 46 publications were included in the qualitative synthesis. Of these, 31 concern the mental health of individuals with a Finnish background in Sweden in general, whereas 15 specifically concern the mental health of Finnish war children and their offspring. One publication is in Finnish (Björklund, 2012), six are in Swedish (Folkhälsomyndigheten, 2019; Kuosmanen, 2001; Lagnebro, 1994; Molarius & Ekholm, 2010; Statens folkhälsoinstitut, 2010; Statens offentliga utredningar, 2017a), and the remaining 39 are in English. Methodology, participant characteristics, and main findings of the 46 publications are summarized in Table 1 (for studies concerning individuals with a Finnish background in Sweden in general) and Table 2 (for studies concerning Finnish war children).

**Table 1.** Summary of Reviewed Publications Concerning Mental Health Among Sweden Finns in General.

Author(s)	Year	Study Design and Population	Main Results
(A) Quantitative Findings			
J. Sundquist	1994	Survey study including 396 Finnish-born and 96 Swedish-born adults (plus groups with other backgrounds).	Finnish-born individuals do not self-report worse mental health than Swedish-born individuals.
Wasserman et al.	1994	Register study including 2748 individuals treated after suicide attempts in five hospitals in Sweden, Finland, Denmark, and Norway.	Suicide attempts were substantially more common among Finnish citizens residing in Sweden, especially among those aged 15–34 years, compared with Swedish citizens as well as to Finns in Finland.
Ferrada-Noli et al.	1995	Register study including 707 cases of violent and unnatural death.	A majority of suicide cases (35 of 60) and undetermined deaths (22 of 35) involve Finnish-born individuals.
Ferrada-Noli et al.	1996	Register study including 202 cases of suicide and undetermined suicide.	Among the cases of undetermined suicide, Finnish-born individuals displayed an elevated alcohol blood concentration compared with Swedish-born individuals (and other groups born abroad).
Ferrada-Noli	1997	Register study including 11,258 cases of violent and unnatural death.	The Finnish-born group is among the groups of people born abroad with the highest suicide rates in relation to their number, with an overrepresentation factor of 2.23.
Johansson et al.	1997	Register study including 95,856 Finnish-born and 3,010,938 Swedish-born individuals >16 years of age (plus groups with other backgrounds).	Finnish-born women and men display elevated suicide rates (RR 1.68 and 2.04, respectively) compared with the Swedish-born population. These suicide rates are higher than those reported for the population in Finland.
Bayard-Burfield et al.	2000	Survey study including 36,890 adult Swedes born in Sweden and other countries (the number of individuals born in Finland is not reported).	Use of benzodiazepines is more common among Finnish-born men (OR 2.24) than Swedish-born men.
Hjern & Allebeck	2002	Register study including 56,076 Finnish-born and 1,313,935 Swedish-born parents, and 73,014 children of one or two Finnish-born parents compared with 1,056,225 children of two Swedish-born parents (plus groups with other backgrounds).	First-generation Finnish-born individuals in Sweden display an elevated suicide risk compared with the Swedish-born population (OR 1.4) and other groups born abroad. Second-generation individuals with two Finnish-born parents display an elevated suicide risk compared with those with Swedish-born parents (OR 1.7); a similar non-significant pattern is seen among those with one Finnish-born parent (OR 1.3).
Iglesias et al.	2003	Survey study including 883 Finnish-born and 17,817 Swedish-born women in the fertile age group (plus groups with other background) during two periods.	Finnish-born women report higher levels of psychosomatic complaints (OR 1.52 and 1.56 for the two periods) than Swedish-born women.
Mäkinen & Wasserman	2003	Register study in which data on 1357 Finnish-born migrants to Sweden >15 years of age who committed suicide during the years 1982–1992 are compared with total population data.	Very high suicide rates (mean 48.2 during the study period) are seen among Finnish-born migrants to Sweden; this is 2× the Swedish mean, 1.6× the Finnish mean, and higher than any other European country. The mean suicide rate for women is 26.5 and for men 76.1; the suicide rate for women has increased by 25% during the study period.
Hjern & Allebeck	2004	Register study including 56,076 Finnish-born and 1,313,935 Swedish-born parents, and 73 014 children of one or two Finnish-born parents compared to 1 056 225 children of two Swedish-born parents (plus groups with other backgrounds) (the same as in Hjern & Allebeck 2002, as far as we can tell).	Alcohol-related morbidity is more common among first-generation Finnish-born migrants (RR 2.1) and among their children with one or two Finnish-born parents (RR 1.9 and 1.6, respectively) compared with Swedish-born individuals and children with two Swedish-born parents.
Hjern et al.	2004	Register study including 56,076 Finnish-born and 1,328,405 Swedish-born parents, and 35,534 children of Finnish-born parents compared with 1056 225 children of Swedish-born parents (plus groups with other backgrounds).	First-generation Finnish-born individuals in Sweden display a higher risk of schizophrenia (RR 1.6) and other psychotic disorders (RR 1.3) compared with Swedish-born individuals. The second generation also displays an elevated risk of schizophrenia (RR 2.5) and other psychotic disorders (RR 1.7) compared with those with Swedish-born parents.
Saraiva Leão	2006	Doctoral thesis encompassing several register studies including between 14,000 and 2.2 million adult Swedes, of whom a varying number are born in Finland or are children of Finnish-born parents (plus groups with other backgrounds).	Reports findings from several studies: • The second generation with Finnish background display higher risks of being hospitalized for a psychotic disorder (HR 2.42), affective disorder (HR 1.39), anxiety disorder (HR 1.61), and personality disorder (HR 2.18) compared with the Swedish majority population. These risks are also elevated compared with second-generation refugees or second-generation migrant workers with other backgrounds. The differences remain after controlling for income and education level. • First-generation women and men are more often affected by schizophrenia (HR 2.48 and 1.56, respectively) and other psychotic disorders (HR 2.31 for both genders) compared with those born in Sweden. Second-generation women and men with two Finnish-born parents are also more often affected by schizophrenia (HR 2.33 and 2.25, respectively) and other psychotic disorders (HR 2.26 and 2.34, respectively) compared with those with two Swedish-born parents. The same goes for second-generation women and men with one Finnish-born and one Swedish-born parent (HR 1.91 and 1.87 for schizophrenia, respectively; HR 1.72 and 1.64 for other psychotic disorders, respectively). • The first and second generations with a Finnish background display the highest risk of alcohol and drug use of all studied groups. Being hospitalized for alcohol or drug use is more common among Finnish-born women (HR 3.64 and 2.33, respectively) and men (HR 3.61 and 2.06, respectively); among women (HR 2.65 and 2.09, respectively) and men (HR 2.47 and 2.19, respectively) with two Finnish-born parents; and among women (HR 1.62 and 1.54, respectively) and men (HR 1.60 and 1.77, respectively) with one Finnish-born parent.
Westman	2006	Register study including 4.4–4.5 million adult Swedes, of whom approximately 160,000–170,000 are born in Finland (plus groups with other backgrounds).	Finnish-born women and men more often make suicide attempts (HR 1.87 for both genders) and display an elevated suicide risk (HR 1.43 and 1.50, respectively) compared with Swedish-born women and men. Finnish-born women are more often hospitalized for a psychotic disorder (HR 1.69), affective disorder (HR 1.20), and anxiety disorder (HR 1.17) than Swedish-born women. Finnish-born men are more often hospitalized for a psychotic disorder (HR 1.24) than Swedish-born men.
Hammar et al.	2009	Survey study including 1083 Finnish-born twin couples, of with either one or both twins have migrated to Sweden.	Male Finnish migrants to Sweden consume less alcohol than those who have stayed in Finland.
Molarius & Ekholm	2010	Survey study in two Swedish counties including 12,280 Swedish-born adults and 976 adults born in another Nordic countries (of which the authors approximate that around 90% are born in Finland).	Alcohol intoxication is more common among those born in another Nordic country compared with the Swedish-born population. No differences regarding mental well-being.
Statens folkhälsoinstitut (Swedish Public Health Agency)	2010	National 5-year survey study including 4387 Finnish-born individuals.	Finnish-born men report fewer days of good mental health, lower levels of mental well-being, and a higher prevalence of current or previous suicidal thoughts compared with the population at large; however, they also report less anxiety.
Arola et al.	2018	Randomized controlled study including 71 Finnish-born individual >70 years of age, of whom 37 participated in an intervention based on group meetings and home visits.	The intervention group displayed a greater sense of coherence after 6 months, but this difference did not remain at 12-month follow-up.
Folkhälsomyndigheten (Swedish Public Health Agency)	2019	Survey study including approximately 10,000 first-, second- and third-generation individuals with a Finnish background in Sweden.	No differences in self-reported overall mental health compared with the total population. Anxiety is less common among those with a Finnish background, but stress is more common and the prevalence of suicidal thoughts or suicide attempts is higher.
Stafford et al.	2019	Register study including 114,393 Finnish-born and 2,606,243 Swedish-born individuals >60 years of age (plus groups with other background).	Elderly Finnish-born individuals are more often affected by a psychotic disorder (HR 1.57) than Swedish-born individuals.
Saarela & Kolk	2020	Register study with Swedish and Finnish data including 4,148,794 Swedes (of whom 5.0% are born in Finland or have one or two Finnish-born parents) and 2,997,867 Finns (of whom 7.3% are born in Sweden or have one or two Swedish-born parents) >17 years of age.	The alcohol-related mortality is higher in Finland than in Sweden. In both Sweden and Finland, individuals with a mixed background (i.e., with on Swedish-born and one Finnish-born parent) display an alcohol-related mortality that is lower compared to the Finnish majority population but higher compared to the Swedish majority population.
Östergren et al.	2021	Register study with Swedish and Finnish data including 104,126 Finnish-born adults in Sweden, 299,033 adult Finns, and 2,033,973 Swedish-born adults.	Finnish-born migrant women in Sweden display an elevated alcohol-related mortality compared with Swedish-born women and similar alcohol-related mortality to women in Finland. Finnish-born migrant men display an elevated alcohol-related mortality compared with Swedish-born men but a somewhat lower alcohol-related mortality than men in Finland.
Östergren et al.	2023	Register study with Swedish and Finnish data including 117,743 Finnish-born individuals in Sweden, 2,453,805 Finns, and 1,706,810 Swedish-born individuals.	Finnish-born migrant men in Sweden display an elevated alcohol-related mortality compared with Swedish-born men, but a lower alcohol-related mortality than men in Finland. However, the longer the Finnish-born men reside in Sweden, the more their alcohol-related mortality approaches that of the Swedish-born male population. Finnish-born migrant women in Sweden also display an elevated alcohol-related mortality compared with Swedish-born women, but a lower alcohol-related mortality than women in Finland. For Finnish-born women in Sweden, alcohol-related mortality does not approach that of Swedish-born women over time. Residing in a neighborhood with a large Finnish-born population does not affect alcohol-related mortality, for either Finnish-born women or men.

(B) Qualitative and Ethnographic Findings			
Ekman et al.	1994	Observational study including nine Finnish-born patients >70 years of age in dementia care.	Although communication with caregivers in dementia care is generally impaired, patients fare markedly better in terms of communication with Finnish-speaking staff members. Openness and responsiveness among staff are also keys to successful communication in this context.
Kuosmanen	2001	Interviews with a strategic sample of 28 Finnish-born men who migrated to Sweden at working age post-1945, of whom 13 are in a marginalized position and 15 are in an integrated position (as comparison group).	The informant narratives focus on experiences and recurrent patterns regarding alcohol abuse and its social consequences, with a particular emphasis on the role of masculinities and health.
Heikkilä	2004	Interviews with 39 Finnish-born individuals >75 years of age with Finnish as their first language, living on their own. Additional 23 interviews with residents, relatives, and staff at a retirement home for Finnish-speaking elderly.	The Finnish language is important for the residents, in healthcare encounters as well as in establishing a safe and familiar everyday environment. Finnish cultural elements, such as food and traditions, are also important. Residents with dementia also clearly seem to be positively stimulated by partaking in the celebration of Finnish holidays and culturally congruent rituals. Relatives are certain that caring for their elderly relatives would have been less successful at a Swedish-language retirement home.
Björklund	2012	Survey study targeting Sweden Finns, with a total of 1796 respondents with good knowledge of the Finnish language.	The generation of Finnish migrants to Sweden who arrived in the 1960s and 1970s are of retirement age, which means that an increasing number need support and care. Individuals born in Finland experience worse mental health than the majority population.
Rosendahl et al.	2016	Interviews with five family members and nine nurses caring for patients with a migration background in dementia care, of whom a family member and one nurse had a Finnish background.	The Finnish language, Finnish food, etc. are vital elements in upholding a safe and familiar everyday environment for patients with dementia.
Söderman & Rosendahl	2016	Interviews with 27 nurses and assistant nurses in dementia care, of whom 12 worked at a Finnish-language facility.	The Finnish language, Finnish food, and Finnish traditions are vital elements in upholding a safe and familiar everyday environment for patients with dementia. Such an environment is more readily established by Finnish-speaking staff.
Statens offentliga utredningar (Swedish Government Official Reports)	2017a	Survey study targeting the national minorities in Sweden, with approximately 2700 respondents (of whom an unknown number are Sweden Finns).	Many Sweden Finns describe how social marginalization and feelings of inferiority have resulted in depression and other psychiatric issues, such as substance use. Many also describe how a limited knowledge of the Swedish language increases the risk of malpractice in healthcare encounters; the risk appears to be most obvious in psychiatry.
Söderman et al.	2018	Observational study at one Finnish-language and one Swedish-language nursing home for individuals with dementia.	A Finnish-language environment creates a sense of community and contributes to better healthcare encounters for individuals with dementia with a Finnish background.

**Table 2.** Summary of Reviewed Publications Concerning the Mental Health Among Former Finnish War Children.

Author(s)	Year	Study Design and Population	Main Results
(A) Mental Health Among Finnish War Children: Quantitative Findings			
Pesonen et al.	2007	Survey study including 410 former war children who have returned to Finland and 1248 non-evacuee individuals of the same age. Based on the Helsinki Birth Cohort Study.	Former war children display 20% more severe depressive symptoms as adults. More former war children display at least mild depressive symptoms as adults (17.7% vs. 10.8%; OR 1.7).
P. Andersson	2011	Survey study including 98 former war children and 63 war veterans (as control group) residing in Sweden and Finland.	Former war children display elevated levels of PTSD symptoms as adults. Fewer former war children display secure attachment patterns (42.9% vs. 57.1%) and more of them display disorganized attachment patterns (26.2% vs. 6.1%).
Räikkönen et al.	2011	Register study including 1719 former war children who have returned to Finland and 11,028 non-evacuee individuals of the same age. Based on the Helsinki Birth Cohort Study.	Former war children display a higher risk of mental illness (HR 1.18), substance-use disorder (HR 1.30), and personality disorders (HR 1.59) as adults. The differences are particularly large among men; among women, a corresponding difference is not observed. For psychotic disorders, affective disorders, and anxiety disorders, no significant differences are observed.
M. Lahti et al.	2012	Register study including 1717 former war children who have returned to Finland and 11,017 non-evacuee individuals of the same age. Based on the Helsinki Birth Cohort Study.	Former war children display an increased risk of being diagnosed with a personality disorder (RR 1.5). This pattern is particularly strong for women.
Alastalo et al.	2013	Survey study including 267 former war children who have returned to Finland and 1536 non-evacuee individuals of the same age. Based on the Helsinki Birth Cohort Study.	No significant differences are observed between groups regarding alcohol use. Male former war children display impaired psychosocial functioning as adults; the same is not seen among women.
Pesonen et al.	2013	Register study including 93 former war children who have returned to Finland and 809 non-evacuee individuals of the same age. Based on the Helsinki Birth Cohort Study.	Former war children display impaired results on various cognitive tests at 20 and 70 years of age. However, no differences are seen regarding cognitive decline over the 50-year period.
N. Santavirta & Santavirta	2014	Register study including 723 former war children who have returned to Finland and 1321 non-evacuee individuals of the same age.	No differences are seen between groups in terms of depressive symptoms or previous depression in adult age.
P. K. Andersson	2015	Survey study including 98 former war children residing in Sweden and 54 controls residing in Finland.	Former war children display elevated levels of PTSD symptoms (RR 10.6) as adults.
T. Santavirta et al.	2015	Register study including 1425 former war children who have returned to Finland and their non-evacuee siblings.	No differences are observed between evacuee and non-evacuee siblings in terms of hospitalization for mental illness in general. Upon analysis of individual diagnoses, female former war children are more likely to have been hospitalized for depression (HR 2.19) compared to their non-evacuee sisters.
J. Lahti et al.	2016	Survey study including 181 former war children who have returned to Finland and 1085 non-evacuee individuals of the same age. Based on the Helsinki Birth Cohort Study.	Former war children display more depressive symptoms as adults. General discussion about the impact of various gene variants.
Heilala	2016	Survey study including 887 former war children living in Sweden or Finland and 1748 non-evacuee controls of the same age.	Former war children do not do worse in terms of psychosocial well-being compared to controls, despite early separation trauma. However, substance use is more common among the former war children. A sense of coherence stands out as an important protective factor. Being separated from the foster family and returning to the family in Finland was stressful for many.
Mattsson*	2018	Survey study including 10 former war children who have resided in Sweden for most of their lives.	Former war children do not display signs of depression as measured by the Beck Depression Scale.
T. Santavirta et al.	2018	Register study including 2992 children of former war children who have returned to Finland and 90,399 children of non-evacuee individuals of the same age.	Daughters of mothers who are former war children display an elevated risk of hospitalization for mental illness (HR 2.04), particularly so for depressive disorder (HR 4.68). Daughters of fathers who are former war children or sons of parents who are former war children do not display corresponding elevated risks.

(B) Mental Health Among Finnish War Children: Qualitative and Ethnographic Findings			
Lagnebro	1994	Interviews with 65 former war children who have resided in Sweden for most of their lives.	The informant narratives bear witness to far-reaching psychological traumatization: in wartime Finland, upon saying goodbye to the biological family, during the journey to Sweden, and in some cases during their time with foster family or at an orphanage. For those who subsequently returned to their biological families, this may also have been a difficult experience. Many were treated well by their foster families; however, others experienced physical and psychological abuse, neglect, and ostracism in Sweden. Even those who have decent experiences with their foster families describe feelings of rootlessness, loss, and guilt. A majority of participants display what is interpreted as various psychosomatic symptoms.
Mattsson*	2018	Interviews with 10 former war children who have resided in Sweden for most of their lives.	All informants have had a decent life in Sweden, but they still experience persistent feelings of emptiness and an ambivalent relationship toward their background as war children. Many also describe anger toward Finland and feeling betrayed. All informants display psychiatric symptoms, such as compulsive behaviors, social anxiety, or phobias.
Y. Andersson et al.	2019	Survey study including 10 former war children who have resided in Sweden for most of their lives.	The informant narratives include many tragic episodes, but the informants do not generally think that their lives have been negatively affected. Many express gratitude for being able to come to Sweden. Feelings of affinity toward and support from other war children are evident. No informants describe serious health-related problems or problems with substance use. The informants do not recall the journey to Sweden, but they describe the separation from their foster families and the return to Finland as painful. Many have struggled with feelings of betrayal and exclusion, which has contributed to a strong determination to succeed in life and work.

*Studies report both quantitative and qualitative findings. HR: hazard ratio; OR: odds ratio; PTSD: post-traumatic stress syndrome; RR: risk ratio.

There are several reasons for presenting the findings concerning the Finnish war children separately. The participants in these studies—individuals who were evacuated to Sweden between 1939 and 1944—have either remained in Sweden as adults or returned to Finland. In the various studies concerning war children, it might sometimes be difficult to determine just how many of the participants have spent a major part of their adult lives in Sweden. Moreover, these studies tend to focus on the impact of separation from caregivers and other adverse childhood experiences, which might not necessarily reflect the experiences of other Sweden Finns. However, it should be noted that the register studies included among the publications concerning the mental health of individuals with a Finnish background in Sweden in general typically include Finnish war children who have remained in Sweden in the overall category of Finnish-born individuals; only those studies that specifically focus on Finnish war children are presented separately.

### The Mental Health of Individuals with a Finnish Background in Sweden in General

Of a total of 31 publications concerning the mental health of individuals with a Finnish background in Sweden in general, 23 are quantitatively oriented (Table 1(A)). A few of these studies focus on specific psychiatric disorders; for example, a comprehensive dissertation shows that first-generation Finnish migrants to Sweden—women and men alike—have a higher risk of developing schizophrenia or other psychotic disorders than the Swedish population at large (Saraiva Leão, 2006). On a group level, women and men in the “second generation” also develop schizophrenia or other psychotic disorders more often, and this holds true for those with two as well as one Finnish-born parent (although the risk appears to be somewhat higher for those with both parents born in Finland). In terms of healthcare use, Finnish-born women in Sweden more often receive inpatient treatment for psychotic disorders, affective disorders (such as major depressive disorder or bipolar disorder), and anxiety disorders compared with Swedish-born women, whereas Finnish-born men more often receive inpatient treatment only for psychotic disorders (Westman, 2006). Likewise, individuals in the “second generation” more often receive inpatient treatment for psychotic disorders, affective disorders, or personality disorders (Saraiva Leão, 2006). These differences remain after adjusting for income and educational level.

A survey study from the Swedish Public Health Agency finds no differences in terms of self-reported general mental well-being among individuals with a Finnish background compared with the population at large (Folkhälsomyndigheten, 2019). In the survey, anxiety is less common among those with a Finnish background, whereas stress is more common. Moreover, individuals with a Finnish background more often report suicidal thoughts or having made a suicide attempt. Notably, suicide and suicidal behaviors are the main focus in 8 of the 23 quantitatively oriented studies reviewed in this section. These studies consistently report a markedly increased risk of suicide among Finnish-born women and men in Sweden (Ferrada-Noli, 1997; Ferrada-Noli et al., 1995; Hjern & Allebeck, 2002; Johansson et al., 1997; Mäkinen & Wasserman, 2003; Wasserman et al., 1994; Westman, 2006). Women and men in the “second generation” with two Finnish-born parents also display an increased risk of suicide compared with those with Swedish-born parents; a similar, albeit non-significant, pattern is seen among those with only one Finnish-born parent (Hjern & Allebeck, 2002). Some of these studies indicate that the risk of suicide among Finnish-born women and men in Sweden is also elevated compared with the population of Finland (Johansson et al., 1997; Mäkinen & Wasserman, 2003). In fact, individuals with a Finnish background in Sweden are one of the most severely affected groups in Sweden in terms of suicide (Ferrada-Noli, 1997; Hjern & Allebeck, 2002). Remarkably, the group also stands out from a European perspective, displaying a higher suicide rate than any single European country (Mäkinen & Wasserman, 2003) (whereas the overall suicide rates of both Sweden and Finland can be considered medium high in a comparison between European countries). However, it should be noted that some of the studies that report particularly high suicide rates among individuals with a Finnish background in Sweden make use of register data from the 1980s and 1990s, and that none of the studies that analyze suicide risk are performed after 2006. Therefore, the current characteristics of the population with a Finnish background in terms of suicidality is unknown.

Eight of the 23 quantitatively oriented studies reviewed in this section focus on substance use. Overall, women and men with a Finnish background in Sweden more often have a high-risk alcohol consumption (as defined by Swedish public health authorities) compared with the population at large, and they have more often used illicit drugs (Folkhälsomyndigheten, 2019; Molarius & Ekholm, 2010). These patterns are seen among the first-generation Finnish migrants as well as among their children in the “second generation” (Hjern & Allebeck, 2004; Saraiva Leão, 2006). Use of benzodiazepines is also more common among Finnish-born men than Swedish-born men in one study (Bayard-Burfield et al., 2000). In terms of healthcare use, Finnish-born women and men, as well as women and men in the “second generation” more often receive inpatient treatment for a substance-use disorder (Saraiva Leão, 2006). Even so, some studies on alcohol use show that individuals with a Finnish background in Sweden tend to consume less alcohol than what is seen among the population of Finland (Hammar et al., 2009; Saarela & Kolk, 2020). Moreover, although Finnish-born individuals in Sweden—women and men—display higher alcohol-related death rates than Swedish-born individuals, these death rates may be somewhat lower than the alcohol-related death rates seen in Finland (Östergren et al., 2021, 2023).

Of the 31 publications concerning the mental health of individuals with a Finnish background in Sweden in general, 8 are qualitatively or ethnographically oriented (Table 1(B)). Five of these studies focus on the care of elderly first-generation Finnish migrants to Sweden with dementia or other neurocognitive disorders (Ekman et al., 1994; Heikkilä, 2004; Rosendahl et al., 2016; Söderman et al., 2018; Söderman & Rosendahl, 2016). Here, the importance of being able to maintain an everyday connection to Finnish culture and traditions and to have the opportunity to speak the Finnish language is consistently highlighted. The elderly informants themselves (a majority of whom are women) as well as their relatives and the care staff working with them experience substantial benefits of a culturally and linguistically congruent care in creating an environment characterized by safety, continuity, and community—not least for those who have lost the Swedish language they had learned as adults because of neurocognitive degeneration. Finnish cultural elements, traditions, and foods clearly contribute to this sense of community; it is noted that even those elderly Sweden Finns who suffer from severe dementia appear to come to life during Finnish holidays and rituals. The informants also describe the presence of Finnish-speaking staff and cooperation with organizations for Sweden Finns as vital parts in achieving culturally competent care.

Only three qualitatively or ethnographically oriented publications concern aspects of mental health other than neurocognitive disorders (Björklund, 2012; Kuosmanen, 2001; Statens offentliga utredningar, 2017a). Two of these are survey studies; the only publication involving in-depth interviews with Sweden Finns is a comprehensive ethnographical dissertation that explores the experiences of Finnish-born labor migrants in marginalized as well as integrated positions in society, with a particular focus on the nexus of high-risk alcohol consumption and masculinity (Kuosmanen, 2001).

### The Mental Health of Finnish War Children

Of a total of 15 publications concerning the mental health of former Finnish war children, 13 present quantitatively oriented findings (Table 2(A)). Several of these studies are based on data from the so-called Helsinki Birth Cohort Study, in which an epidemiological cohort of women and men born in Helsinki, Finland in the years 1934–1944 have been followed using register data as well as subsequent clinical assessments of a smaller cohort subgroup. This design makes it possible to compare the health status over time among those individuals who were evacuated to Sweden during the Winter War and Continuation War compared with those of similar age who remained in Finland.

Research findings on the mental health of former Finnish war children are diverse and sometimes contradictory. Several studies point to an increased prevalence of various forms of mental distress among former war children: two studies report elevated levels of depressive symptoms in adult age (J. Lahti et al., 2016; Pesonen et al., 2007), and there are also indications of a higher risk of developing post-traumatic stress disorder (P. Andersson, 2011, P. K. Andersson, 2015), personality disorders (M. Lahti et al., 2012; Räikkönen et al., 2011), or substance-use disorders (Heilala, 2016; Räikkönen et al., 2011). However, one study finds no differences in terms of prevalence and symptoms of major depressive disorder when former war children are compared with a same-age control group (N. Santavirta & Santavirta, 2014). Furthermore, no differences between evacuated and non-evacuated siblings are seen in terms of inpatient treatment for mental illness, although upon subgroup analysis, former evacuee women have more often received inpatient treatment for major depressive disorder compared with their non-evacuated sisters (T. Santavirta et al., 2015). In a large survey study, former war children do not report worse psychosocial outcomes than controls, in spite of the occurrence of early separation trauma and difficulties upon reuniting with the family in Finland; here, sense of coherence is highlighted as a hypothetical protective factor (Heilala, 2016).

We have identified only one study concerned with the mental health among children of former Finnish war children (T. Santavirta et al., 2018) This study reports that daughters whose mothers are former war children have more often been admitted to psychiatric inpatient treatment because of mental illness in general and major depressive disorder in particular, whereas a similar tendency is not seen among daughters whose fathers are former war children or among the sons of former war children.

Of the 15 publications concerning the mental health of former Finnish war children, three present qualitatively or ethnographically oriented findings (Table 2(B); thus, one study reports both quantitative and qualitative findings). Here too, the findings are mixed. In one study, former Finnish war children bear witness to far-reaching traumatization during the war in Finland as well as upon leaving their families, during the journey to Sweden, and in some cases also during their stay in a foster family or at an orphanage in Sweden (Lagnebro, 1994). Those war children who subsequently reunited with their parents in Finland also describe this as complicated. Many war children report being treated well in their foster families, whereas others experienced physical and psychological abuse, neglect, and ostracism in Sweden. Notably, even those who were treated properly by their foster parents describe feelings of homesickness, rootlessness, and guilt. Most of the informants in this study also display what is interpreted as various psychosomatic symptoms.

In yet another study, most of the informants have created decent lives for themselves as adults in Sweden, but they still report persistent feelings of emptiness and ambivalence with respect to their backgrounds as war children (Mattsson, 2018). Many also experience anger toward their birth country Finland, associated with feelings of being deserted. All informants in this study display psychiatric symptoms, such as compulsive behaviors, social anxiety, or phobias. Lastly, the informants in a third study describe a number of tragic episodes in their lives; in spite of this, however, they still believe that they have not been negatively affected overall (Y. Andersson et al., 2019). Many of them express gratitude toward Sweden, describe a great affinity toward other former war children, and view this group as a source of emotional support. None of the informants in this study report any severe mental health issues or substance use problems. They do describe the separation form the foster family and return to Finland as painful and several of them have struggled with feelings of betrayal and of not belonging, which have resulted in a strong urge to get revenge by succeeding in their professional lives.

### Assessment of Publication Bias

Searches on ClinicalTrials.gov, the WHO International Clinical Trials Registry Platform, the EU Clinical Trials Register, and the Open Science Framework did not reveal any previous or ongoing registered studies that have not published results. This indicates that the risk of publication bias is low.

## Discussion

The reported pattern of findings and trends synthesized in this scoping review shows that Finnish-born women and men in Sweden tend to be worse off in terms of mental health compared with the Swedish-born majority population. In most of the reviewed register studies, this holds true even after adjusting for socioeconomic factors. Finnish-born individuals in Sweden are more often diagnosed with various serious psychiatric disorders, such as schizophrenia and other psychotic disorders. Alcohol and other substance-use disorders are also more common among Finnish-born women and men. Several studies also report substantially higher suicide rates in this group—notably, the observed suicide rates of the Finnish-born population in Sweden are among the highest recorded rates for any foreign-born group in Sweden and also stand out from a European perspective, although no recent studies exist.

The reviewed findings concerning the mental health of former Finnish war children are more equivocal. Some studies report a higher prevalence of post-traumatic stress disorder and major depressive disorder in this group, whereas other studies find no such differences. In qualitative studies, most interviewed former war children describe numerous traumatic experiences during wartime and evacuation. Not least for those who subsequently reunited with their families in Finland, separation from the Swedish foster family—which, of course, occurred at an older age than the original separation from the Finnish family—and going back to studying in a language they had partly forgotten appear to have been difficult experiences. However, no distinct patterns in terms of psychiatric and psychosocial problems have been found among the former war children.

Mental health among children of Finnish-born individuals in Sweden—the so-called “second generation”—is also, on a group level, worse than what is seen among children of the Swedish-born majority population. Individuals in Sweden with one or two Finnish-born parents receive more psychiatric inpatient treatment, more often have a harmful level of use of alcohol and illicit drugs, and display higher suicide rates (the last finding mostly apply to those with two Finnish-born parents). Compared with first-generation Finnish migrants, however, the mental health of the “second generation” is generally somewhat better. It should be noted that the studies on the “second generation” reviewed here almost exclusively describe their mental health in adulthood; there is, apparently, very little research about the mental health of children and adolescents with a Finnish background in Sweden. We have not been able to draw any conclusions regarding the “third generation”, i.e., grandchildren of Finnish migrants; at least one of the reviewed studies planned to assess the health status of this group, but ultimately reached too few individuals (Folkhälsomyndigheten, 2019).

In sum, the reported pattern of findings and trends indicates that a substantial mental health gap exists between the minority population with a Finnish background in Sweden and the Swedish majority population. Even so, some of the studies that also involve comparisons with Finns in Finland indicate that people with a Finnish background in Sweden hold an intermediate position in terms of mental health: it is generally worse than that observed among the Swedish majority population, but in some respects also somewhat better than that reported in Finland. For example, alcohol intake and alcohol-related mortality among individuals with a Finnish background in Sweden are lower than among Finns in Finland. A reasonable interpretation of these findings is that Finnish migrants to Sweden have, in some respects, actually improved their mental health status compared with what would, on a group level, have been the case if they had remained in Finland. Still, the mental health of people with a Finnish background in Sweden obviously lags behind that of the majority population as well as that of many other groups with a migration background, at least in terms of suicide rates.

Because a large part of the population with a Finnish background in Sweden came to the country as labor migrants in the post-Second World War period, comparisons with other groups of labor migrants to Sweden and their offspring might be relevant. Besides labor migration from the Nordic countries and Germany, large groups from Italy, former Yugoslavia, Greece, and Turkey came to Sweden in the 1960s and 1970s to meet the demand for labor in the same industrial sectors as the Finns (Byström & Frohnert, 2017). Unfortunately, the experiences of these groups have been less studied, although there is a small research literature on Italian post-war migration to Sweden (Cavallin, 2015; Granberg, 1991; Järtelius, 1987). With the exception of a small number of register studies that report health outcomes for Italians, Greeks, ex-Yugoslavs, and Turks in Sweden, these groups are often treated as one—“Southern Europe”—in which other migrant groups are also included. Moreover, many in the ex-Yugoslav group came as refugees during the 1991–2000 Yugoslav Wars, and their mental health is typically affected by traumatic experiences that are not necessarily shared by those ex-Yugoslavs who came as labor migrants in earlier decades. In some studies, the mental health among ex-Yugoslavs in Sweden is indeed worse than what is observed among both the Swedish-born and the Finnish-born parts of the population (Saraiva Leão, 2006). Migrants from Southern Europe also have an increased risk of psychotic disorders compared with the non-migrant population, similar to that found in most migrant groups (see below), but their mental health in terms of suicide rates, psychosocial well-being, etc. is generally better than or similar to that of the Swedish-born reference group (Ferrada-Noli, 1997; Hjern & Allebeck, 2002, 2004; Johansson et al., 1997; Saraiva Leão, 2006; Westman, 2006).

### Potential Mechanisms Behind the Patterns

Available research offers little insight into the causal mechanisms behind the observed patterns of impaired mental health among individuals with a Finnish background in Sweden. A majority of the reviewed register studies use a cross-sectional design, which does not allow for analysis over time. With the exception of the former Finnish war children who are included in the Helsinki Birth Cohort Study, we did not identify any longitudinal studies concerning the mental health of individuals with a Finnish background in Sweden. Any attempts to explain the reasons behind the observed patterns must therefore be regarded as hypothetical. For example, a tendency for high-risk alcohol consumption based on a particularly strong drinking culture may of course lead to the development of psychiatric problems such as major depressive disorder; on the other hand, alcohol intake could also be viewed as a form of self-medication in the face of various forms of pre-existing distress due to traumatic war experiences, etc. Naturally, this causal uncertainty is not unique for Finnish migrants to Sweden. It is well established that migrants and their offspring have a higher risk of developing schizophrenia and other psychotic disorders, but the exact reasons behind this pattern have been the subject of long debate (Dykxhoorn & Kirkbride, 2019; Morgan et al., 2019). To date, research has ruled out some suggested causal pathways, such as the idea that individuals with a high inherent risk of psychosis tend to migrate more often or that obstetric complications or early infections that are more common in low-income countries might explain the observed differences. Most evidence points to the higher levels of stress pre-migration, during migration, and post-migration as the main explanatory factor behind the increased risk of psychotic disorders among migrants (Dykxhoorn & Kirkbride, 2019).

A closely related concept is that of weathering, which describes cumulative health effects of stressors such as poverty, precarious work, substandard housing, neighborhood unsafety, racism, and discrimination (Geronimus, 1996). Hypothetically, weathering as a phenomenon can explain some of the negative health outcomes observed in individuals with a Finnish background in Sweden. Finnish labor migrants were typically seen as good workers, but they were also subjected to negative stereotypes and there is evidence of far-reaching discrimination against the Finnish migrants and their children (Ågren, 2006; Beckman, 2018; Kuosmanen, 2001; Liimatainen, 2022; Lukkarinen Kvist, 2006; Weckström, 2016). Many Finnish labor migrants have described their lives in Sweden during the 1960s and 1970s as fun, exciting, and characterized by a sense of community within the group, but research has also revealed a more disheartening picture of marginalization and of having to hide one's Finnish roots to fit in and be accepted in society. Moreover, although most Finnish migrants to Sweden are white (for an in-depth discussion of whiteness as a sociocultural entity, see Dyer, 1997), prevailing pseudoscientific ideas about biological race have tended to depict Finns as racially inferior to the Swedish majority population (Liimatainen, 2022). Also, because of intermarriage between different migrant groups, it has been estimated that approximately 7% of “second-generation” Sweden Finns are of mixed racial heritage and might be racialized as persons of color (Beckman, 2018). The potentially detrimental effects of racism and racial discrimination on various health outcomes are by now well established (Paradies et al., 2015; Williams et al., 2019) and might have a particularly negative impact on this subgroup of Sweden Finns.

Various barriers to healthcare might also negatively affect the mental health of individuals with a Finnish background in Sweden. Such barriers include language difficulties, cultural barriers (e.g., stigma and differences in symptom presentation or help-seeking tendencies), and structural barriers (e.g., economic hardship, difficulties navigating the healthcare system, discrimination, and racism) (Metzl & Hansen, 2014; Nutbeam & Lloyd, 2021; Pemberton et al., 2019; Wångdahl, 2017) Several studies reviewed here show that individuals with a Finnish background in Sweden more often receive psychiatric inpatient treatment for problems such as psychotic disorders or substance-use disorders. Paradoxically, however, more frequent use of inpatient services may actually reflect lower access to adequate outpatient treatment: if patients had received help from primary care or psychiatric outpatient services in time, they might not have needed subsequent hospital admission. Other migrant groups in Sweden tend to have lower access to adequate treatment compared with the Swedish-born population, which may in turn lead to an increased need for emergency- and inpatient-level care (Gubi et al., 2022; Hollander et al., 2020; Terhune et al., 2020). Available research does not tell us if this patterns is also valid for the group with a Finnish background.

Given the consistent overall picture of worse mental health among individuals with a Finnish background in Sweden reflected in the quantitatively oriented research reviewed here, it is somewhat surprising that the health-related experiences within this population have not been more thoroughly explored through qualitative studies. Most of the qualitatively and ethnographically oriented studies that we have identified in this scoping review concern Finnish-language care for elderly Sweden Finns with dementia and other neurocognitive disorders. This field of research highlights obvious advantages in terms of quality of life when the Finnish language and Finnish culture—e.g., foods and traditions—are allowed to become a central part of everyday care. Examples of culturally congruent activities described in these studies are the incorporation of Finnish songs and music into daily life, the celebration of the Finnish Independence Day and Finnish Mother's Day, cooking and serving traditional holiday dishes (such as Karelian pasties, carrot and rutabaga casseroles, and herring salad), and hot sauna baths. Not least for those elderly care recipients with advanced dementia who might have lost competences acquired in adult age, such as Swedish language proficiency (Ellajosyula et al., 2020), culturally and linguistically competent care can ensure a crucial sense of continuity and safety in everyday life. Regarding other areas of mental health, more qualitative research is needed to explore causal pathways and potential preventive measures.

### Perspectives on Sweden Finn Identity

This scoping review also underscores the somewhat ambiguous nature of the term Sweden Finns. Very few of the reviewed studies use this terminology, and when they do it is often uncertain whether it is applied correctly—typically, the term Sweden Finns is used to denote the entire population with a Finnish background in Sweden, without ever asking whether those included view themselves as Sweden Finns in accordance with the principle of self-identification described above. Most of the studies reviewed here make use of register data to investigate the group-level health status of Finnish-born individuals or their children in the “second generation”, but it is, of course, not possible to tell whether these individuals actually self-identify as Sweden Finns. Notably, members of the Swedish-speaking minority in Finland who have moved to Sweden will also be included in the Finnish-born group in these studies. In Swedish research, because of a fear of stigmatization combined with a national self-image as a post-racial society, it is not customary to ask about race and/or ethnicity in the way that is usually taken for granted in the United Kingdom or the United States, which makes it difficult to explore various forms of inequality (Wikström & Hübinette, 2021). For some research on the health of migrant groups, register data on birth country and parents’ birth country can be used as proxies for ethnicity or race; for the five officially recognized national minorities in particular, however, this option is typically not available, because many of those who belong to a national minority have lived in Sweden for several generations (Manga et al., 2022).

Researcher Tuire Liimatainen (2022) describes five different discourses related to the concept of Sweden Finns among individuals with a Finnish background in Sweden:

- Within a diasporic discourse, the Sweden Finn is first and foremost a labor migrant and an “outsider” in Sweden; someone who tries to keep a Finnish identity alive in exile, although this identity can become fixed and rigid as the years go by, not reflecting cultural change that has occurred in Finland since the time of migration.
- In a transnational discourse, coming to Sweden is more of a voluntary life choice and one's identity can more readily embrace both Finnishness and Swedishness. This attitude might be more common among younger Sweden Finns who have come to Sweden to pursue a higher education rather than to find manual labor.
- A hyphenated discourse reflects a hybrid existence in which Finnishness and Swedishness are building blocks that are combined with other elements, such as gender, social class, sexual orientation, etc., to create a multifaceted cultural identity (for example, “rainbow migration” of individuals seeking a more tolerant attitude toward LGBTQI + individuals in Sweden has been reported) (Hoppu, 2021). This discourse might be more common among second- and third-generation Sweden Finns, who often have no personal experience of life in Finland.
- Within a minority discourse, being a Sweden Finn is a unique position—a specific identity with a specific history rather than simply a mix of Finnishness and Swedishness. This attitude has usually been the starting point for political struggle for minority rights in Sweden. It might also appeal more to those second- and third-generation Sweden Finns who have not learned the Finnish language; this might hinder a full identification as Finn, but being a Sweden Finn is still an available option.
- A final variant is an autochthonous discourse, in which Sweden Finns are not primarily migrants or newcomers but a group with far-reaching historical roots in Sweden.

This variety illustrates the many existing perspectives on Sweden Finns in society and in research—a variety that inevitably gives rise to ambiguity and contradictions. A diasporic discourse, with a focus on the Sweden Finn as labor migrant, appears to have dominated in research, and only recently has a minority discourse centered around self-identification surfaced. A transnational view, however, is lacking in the research literature; very little research has explored health and health-related experiences among later, more highly educated Finnish migrants to Sweden.

Finally, it can be noted that there is very little published research concerning the task of improving the mental health of Sweden Finns and others with a Finnish background in Sweden through treatment interventions or preventive measures targeting the group. Naturally, general advice and guidelines apply to this population too, but it is also very likely that interventions tailored specifically to the needs of the group could play an important role in improving health and well-being. It is reasonable to assume that the distinct epidemiological patterns reported in the research reviewed here could have led to some form of public health interventions to strengthen outreach and improve uptake, but this does not seem to have happened to any significant extent. An exception is the qualitative research about the care of Finnish-born elderly people with dementia and other neurocognitive disorders, where the importance of cultural and linguistic competence for creating safety, continuity, and meaning in everyday life has been underscored. Similar to the Swedish Public Health Agency (Folkhälsomyndigheten, 2019), we want to highlight the necessity of working toward the identification of the causal pathways behind the reported substantial differences in mental health between the population with a Finnish background in Sweden and the Swedish population at large, so that a tailored response can be designed. Moreover, some available statistics—such as suicide rates—are based on fairly old epidemiological data and should be updated using more recent register data. Lastly, we again wish to emphasize the need for qualitative research in which Sweden Finns and others with a Finnish background in Sweden are allowed to voice their healthcare experiences, needs, and expectations, so that a nuanced understanding can be achieved and potential paths forward can be identified.

## Conclusion

The reported pattern of findings and trends synthesized in this scoping review indicates that a substantial mental health gap exists between the minority population with a Finnish background in Sweden and the Swedish majority population, even after adjusting for socioeconomic factors. Perhaps most notably, several studies report substantially higher suicide rates among individuals with a Finnish background in Sweden (although there is a need to update these research data to include more recent years). In contrast to other groups that arrived in the country as labor migrants in the post-Second World War period, the population with a Finnish background in Sweden does not display a healthy migrant effect, at least not when it comes to mental health. The reasons behind this pattern are not clear and are most likely complex. Even though many Finnish migrants have described their lives in Sweden as fairly good, there is certainly a long history of marginalization and discrimination against Finns in Sweden, which might negatively impact their health and well-being. Moreover, many in the Finnish war and post-war generations were affected by various types of war trauma and poverty in a way that the Swedish majority population was generally not. However, neither experiences of discrimination nor war trauma are unique to the Finnish group compared with other migrant populations in Sweden. There are possibly further contributing historical factors that emanate from the centuries-long, largely asymmetrical relationship between Sweden and Finland. As a complement to the mostly quantitative data reviewed here, there is an urgent need for in-depth qualitative and cross-disciplinary research that explores the health-related experiences of the Sweden Finnish population and the potential mechanisms behind the observed mental health gap.

## Notes

1. The Winter War (1939–1940) and the Continuation War (1941–1944) were two campaigns of the Second World War against the Soviet Union on Finnish land. In Finland, these particular terms (rather than the broader “Second World War”) are commonly used.
